# Expression pattern of ZNF33B in bovine ovaries and the effect of its polymorphism on superovulation traits

**DOI:** 10.5194/aab-65-69-2022

**Published:** 2022-02-22

**Authors:** Changhong Li, Peijun Xia, Yijuan Ma, Xinyue Zhang, Yijia Liu

**Affiliations:** 1 College of Life Sciences, Baicheng Normal University, Baicheng, Jilin, China; 2 College of Animal Science, Jilin University, Changchun, Jilin, China

## Abstract

ZNF33B belongs to recently duplicated Krüppel-associated box domain zinc finger proteins (KRAB-ZFPs), which is widely present in various organs, and some evidence showed that its expression is altered in the ovary undergoing superovulation. In this study, the expression of ZNF33B in ovary and early embryo was determined by immunohistochemistry and immunofluorescence techniques. Results showed that the expression of ZNF33B in the ovary was mainly in the cytoplasm of oocytes and granulosa luteal cells of ovarian corpus luteum and significantly reduced during follicular ovulation to luteal degeneration. The expression of ZNF33B in the early embryo transferred from the nucleus to the whole cell, suggesting that the expression of ZNF33B is spatiotemporally specific. Then, in combination with the single nucleotide polymorphism (SNP) database, the g.-61G > T mutant of the 5 ${}^{\prime }$ -untranslated region (5 ${}^{\prime }$ UTR) of the ZNF33B gene was screened out from 556 Changbaishan black cattle, and the frequency of the mutant gene was counted. The statistics of superovulation and superovulation traits confirmed significant differences between the two genotypes in the quantity and quality of oocytes obtained after superovulation. This study confirmed, for the first time, the effect of ZNF33B gene polymorphism on superovulation traits and suggested that the mutation could provide a basis for cattle breeding and improving animal fertility.

## 1 Introduction

Zinc finger proteins were first found in Xenopus oocytes, which are widely distributed in organisms (Krishna et al., 2003). Nearly 1 % of the sequences in human genome encodes proteins with zinc finger structure. Zinc finger structure can specifically bind to the DNA and RNA sequences of some molecules (Groner et al., 2007) or to itself and other zinc finger proteins to regulate gene expression at transcription and translation levels (Hand et al., 2007). C ${}_{2}$ H ${}_{2}$ zinc finger protein accounts for approximately 53 % of the mammalian genome transcription factor spectrum (Iuchi et al., 2001; Agarwal et al., 2007). More than 30 % of human C ${}_{2}$ H ${}_{2}$ zinc finger proteins have Krüppel conserved box structure, belonging to Krüppel-associated box domain zinc finger proteins (KRAB-ZFPs) (Mark et al., 1999). The KRAB family could be divided into at least three subtypes: KRAB (A) with only a KRAB-A box, KRAB (AB) with a traditional KRAB-A box and traditional KRAB-B box, and KRAB (Ab) with a traditional KRAB-A box and a highly divergent KRAB-B box (Ecco et al., 2017). The protein size and amino acid sequence of KRAB box structure and zinc finger binding domain at the C terminal differed among different subtypes (Iuchi et al., 2001; Yang et al., 2017). During the early stages of embryogenesis, KRAB-type zinc finger proteins could induce specific silencing of endogenous reverse transcription elements by mediating histone methylation, histone de-acetylation, and DNA methylation, resulting in changes in chromosomal epigenetic modifications. In this manner, genomic stability and normal embryonic development could be maintained (Rowe et al., 2011). In addition, KRAB zinc finger protein can bind to target DNA by fusing a heterologous nucleic-acid-binding domain, thus playing the role of transcriptional inhibition (Abrink et al., 2001; Agaewal et al., 2007). ZNF33B belongs to the KRAB (AB) zinc finger protein, and it has two modules of the Krüppel conserved cassette, the KRAB-A box and the KRAB-B box. ZNF33B has the potential to play a regulatory role in early embryonic development through the unique regulation of the KRAB zinc finger protein (Bellefroid et al., 1991). ZNF33B also has the potential to maintain normal early embryonic development through KRAB zinc-finger-protein-specific regulation.

A study of the structure, genetic sequence, and transcription of the 1.4 megabase pair (Mb) cluster of DNA gene sequences linking the pericentromeric satellites of human 10p11 revealed the presence of four zinc finger genes in proximal 10p11: ZNF33A, ZNF37A, ZNF25, and ZNF248, while ZNF33B is located in its opposite 10q11 (Tunnacliffe et al., 1993); all of these are C ${}_{2}$ H ${}_{2}$ KRAB zinc finger genes containing CpG islands at the 5 ${}^{\prime }$ end and are widely found in adult tissues. ZNF33A and ZNF33B are located 230 kilobase pairs (kb) apart at 10P11.2 and 10q11.2 within chromosomes, with ZNF33A located in cluster A and ZNF33B in cluster B (Tunnacliffe et al., 1993). Both have a large number of unique expressed sequence tags (ESTs) (Guy et al., 2000), complete open reading frames and KRAB frames, and zinc finger regions (Bellefroid et al., 1993; Tunnacliffe et al., 1993). Seven of the 16 zinc fingers of ZNF33A and ZNF33B are different; comparison of the zinc finger structures of this segment in marmosets, sloths, pigs, and giant whales by sequencing showed that ZNF33B had more similarity between species, indicating that it is closer to the original ZNF33 gene, while ZNF33A was significantly different among species and may recognize different target sequences (Guy et al., 2003).

The expression of ZNF33B as a transcription factor (Zhou et al., 2010) was differentially expressed in the supernumerary ovulation and normal ovulation groups, with significantly higher expression levels in the normal ovulation group than in the supernumerary ovulation group (De Los Santos et al., 2012). Differences in ZNF33B levels exist within the follicular fluid between the supernumerary ovulation group and the normal ovulation group, and the expression levels of ZNF33B within the oocytes change accordingly (De Los Santos et al., 2012).

The effect of ZNF33B on oocyte development has not been reported before, but previous studies have shown that it may have a certain effect on oocyte maturation in the stimulation of superovulation (De Los Santos et al., 2012). Therefore, the present study aimed to determine and understand the effect of ZNF33B polymorphism on the superovulation of cattle by studying the expression and location of ZNF33B in ovary and early embryo and the ovulation of different genotypes under the stimulation of superovulation. It also aimed to provide the mechanism for analyzing the molecular regulation pathway and its role on zinc finger protein and the maturation of oocytes. The theoretical basis is provided for improving the reproductive power of female animals and early selection and breeding.

## 2 Materials and methods

### 2.1 Animals and sample collection

All animal tissues used in the experiments were obtained from Changbaishan black cattle raised in the beef cattle farm of Jilin Black Hairy Cattle Industrial Group Co. (Changbaishan black cattle are beef cattle developed via hybridization of Japanese black cattle with local cattle). All the ovaries were collected from the slaughtered cattle on the day of slaughtering. This source of ovary material represents a byproduct of the food industry, and it is more readily acceptable than euthanasia of animals specifically for scientific purposes.

Bilateral ovaries were removed within 20 min after the cattle were slaughtered. Some ovaries were stored in 0.9 % NaCl solution with 1 % penicillin-streptomycin at 37 ${}^{\circ }$ C for collecting oocytes and granulosa cells, and some were directly trimmed into suitable shapes for the paraffin section. The last part of the ovary was directly stored in liquid nitrogen for protein extraction.

### 2.2 Western blot analysis

Proteins in the cortical, medullary, and corpus luteum of the ovary were extracted using radioimmunoprecipitation assay (RIPA) lysis buffer (Biosharp, BL504A), and protein concentration was measured with a Bradford protein assay kit (Sangon Biotech (Shanghai), C503031). Total proteins (10 µ g per lane) were separated with sodium dodecyl sulfate-polyacrylamide gel electrophoresis and transferred to the cellulose acetate membrane. The membrane was blocked in 10 % skimmed milk powder and then incubated with Rabbit ZNF33B polyclonal antibody (MyBioSource, MBS9406181, 1:1000 ). Membranes were washed three times and incubated with Goat Anti-rabbit IgG H&L (HRP) pre-adsorbed (Abcam, AB7090) in a 1:2000 dilution. Afterwards, proteins were detected using a pro-light HRP chemiluminescence kit (Tiangen Biotech, PA112) in accordance with the manufacturer's instructions. A GAPDH antibody (Santa Cruz Biotechnology, sc-47724, 1:2000 ) was used to monitor sample loading. The blots were quantified using ImageJ (v1.48, National Institutes of Health).

### 2.3 Immunohistochemistry

The ovaries were dissected and fixed in 4 % neutral formalin, dehydrated, and embedded in paraffin, and then serial sections were made. Immunohistochemistry analysis was conducted on the sectioned ovaries to determine the spatial distribution of ZNF33B. The sliced samples were boiled in citric acid sodium citrate buffer (0.02 M; pH 6) in an induction cooker for 10 min to recover antigens. Then, the samples were cooled to room temperature. The samples were incubated with 3 % hydrogen peroxide for 30 min to block endogenous peroxidases and then incubated with Rabbit ZNF33B polyclonal antibody (MyBioSource, MBS9406181, 1:100 ). The slides were washed three times with phosphate-buffered saline (PBS) and incubated with pre-adsorbed HRP (Abcam, AB7090). The immunohistochemistry reaction could be identified using the DAB chromogenic reagent kit (Maixin Biotech, DAB-0031) and counterstained with hematoxylin staining solution. Normal non-immune serum was used as the negative control.

### 2.4 Collection of bovine oocytes and in vitro maturation

Follicular fluid was extracted from 2–8 mm normal follicles on the surface of the ovaries and transferred into HEPES buffer, which includes 10 Mm HEPES, NaCl, NaH ${}_{2}$ PO ${}_{4}$ , penicillin, and 1 % PVA (Sigma, P1763). Cumulus–oocyte complexes (COCs) were aspirated into a clean HEPES buffer containing 1 % PVA. The COCs were washed twice with in vitro maturation (IVM) medium, which consisted of Medium 199 (Gibco, 12340) containing 10 % fetal bovine serum (Gibco, 10099141), 1 % penicillin/streptomycin sulfate solution (Sigma, 516104), 0.1 g L ${}^{-1}$ sodium pyruvate (Sigma, P4562), 10 µ g mL ${}^{-1}$ β -estradiol (Sigma, E2758), 10 µ g mL ${}^{-1}$ follicle-stimulating hormone (FSH), and 0.6 mM L-cysteine (Sigma, C7352). They were quantified and collected in drops of culture medium for in vitro oocyte maturation. Then, 18–20 COCs were placed in each drop and transferred to a 38.5 ${}^{\circ }$ C incubator with 5 % CO ${}_{2}$ for 24 h.

### 2.5 Acquisition of early embryos

Early embryos were obtained by in vitro fertilization. Frozen semen (Simmental semen from the Yanbian Animal Husbandry Development Group Co., Ltd.) straws were quick thawed in a 37 ${}^{\circ }$ C water bath, and then the thawed semen was transferred to equilibrated Dulbecco's phosphate-buffered saline. After the semen was gently shaken, it was centrifuged for 3 min at 1300 rpm, and this process was repeated two times. The semen was resuspended and centrifuged for 3 min at 1300 rpm. The precipitated semen was transferred to a straw containing equilibrated in vitro fertilization solution, and it was subsequently placed in an incubator at 38.5 ${}^{\circ }$ C with 5 % CO ${}_{2}$ for 30 min. Then, the liquid was collected from the upper layers and inspected with a microscope to calculate sperm viability and density. After being washed with in vitro fertilization solution, the mature COCs and the sperm were transferred to equilibrated fertilization drops; each of these droplets contained 15 COCs, and they were placed in an incubator at 38.5 ${}^{\circ }$ C with 5 % CO ${}_{2}$ for fertilization. After 24 h, the cumulus cells were removed using 0.1 % hyaluronidase (Sigma, H3506) enzyme solution.

### 2.6 Immunofluorescence detection

Early embryos at different stages were placed in 24-well Petri dishes containing 4 % paraformaldehyde and fixed at room temperature with ventilation for 30 min. After fixing, the embryos were permeabilized with 0.5 % Triton X-100 at 4 ${}^{\circ }$ C for 20 min. Then, the early embryos were transferred to a 1 % Bovine serum albumin (BSA) solution and incubated at 37 ${}^{\circ }$ C for 1 h. The Rabbit ZNF33B polyclonal antibody (MyBioSource, MBS9406181) was diluted 200 times with 1 % BSA solution, followed by incubation with the samples overnight at 4 ${}^{\circ }$ C. The fluorescein isothiocyanate (FITC)-conjugated AffiniPure Goat Anti-rabbit IgG (Boster, BA1105) was also diluted with 1 % BSA solution, followed by incubation in the dark with the samples for 1 h in the incubator at 37 ${}^{\circ }$ C. Next, the samples were incubated with DAPI (Sigma, St. Louis, MO, USA) fluorescence staining reagent and diluted 1000 × for 2 min at room temperature. Then, the slides were sealed, and the expression patterns of the target proteins were observed. The fluorescence staining intensity of early embryos at different developmental stages was detected with fluorescence intensity analysis software. In the abovementioned immunofluorescence staining process, the samples were repeatedly washed at each step.

### 2.7 Superovulation

A total of 556 Changbaishan black cattle were randomly selected from the beef cattle farm of Jilin Black Hairy Cattle Industrial Group Co. These cattle were treated for superovulation by using the protocol provided by the AnBo Embryo Biotech Center (Beijing, China). Superovulation was induced by the 16 d FSH–CIDR (EAZI BreedTM CIDR (progesterone), cattle insert) – prostaglandin (PG) – luteinizing hormone-releasing hormone method (Deng et al., 2015). Each uterine horn was washed with 500 mL phosphate buffer to obtain early embryos. The embryos were immediately treated with PG F2 alpha and washed by an embryo filtration device. The embryos were observed under the microscope to detect the score of embryonic quality.

**Figure 1:**
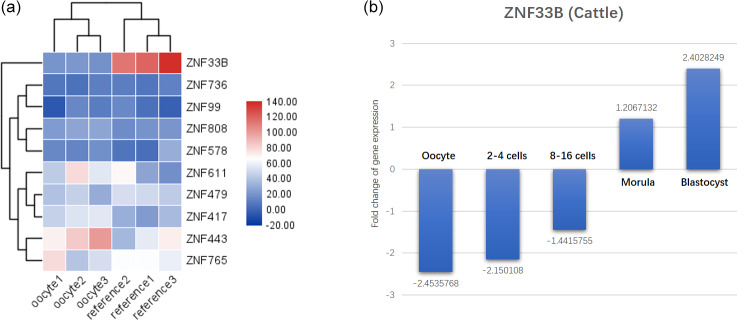
Identification results of recently duplicated genes in KRAB-ZNFs during oocyte development. **(a)** Analysis of 10 rapidly differentiating genes in human oocytes and significantly decreased expression of ZNF33B. **(b)** ZNF33B was found to be consistently poorly expressed at the oocyte and 2–16 cell stages in transcriptomic data from developing bovine embryos, but the expression was gradually upregulated. Upon entering the mulberry embryo and blastoderm stage, ZNF33B was in a high expression state.

**Table 1:** Sequence of the primer.

Sequence		Size (bp)	Annealing temperature
F	GGAAGTTAAACAGCCTAGT	620	54 ${}^{\circ }$
R	GGTTCTGTTCTTGATTTGC		

“bp” indicates a base pair.

### 2.8 DNA extraction

A 10 mL blood sample was collected from the bovine jugular vein and placed into an anticoagulant tube containing the anticoagulant EDTA. Genomic DNA was extracted in accordance with the AxyPrep blood genomic DNA kit (Axygen, AP-96-BL-GDNA-4).

### 2.9 Genotyping

For the genotyping of ZNF33B gene polymorphism, the primers in Table 1 were designed on the basis of ZNF33B gene sequence (gene ID: 520684). ZNF33B was amplified in the polymerase chain reaction (PCR) instrument (BIO-RAD) by using the following PCR protocol: first, it was pre-denatured at 94 ${}^{\circ }$ C for 2 min and then denatured at 94 ${}^{\circ }$ C for 30 s, annealed at 54 ${}^{\circ }$ C for 30 s, and extended at 72 ${}^{\circ }$ C for 40 s. After 30 cycles, a final extension at 72 ${}^{\circ }$ C for 10 min was performed, and ZNF33B was finally stored at 4 ${}^{\circ }$ C. PshA I restriction enzyme was used to digest PCR products at 37 ${}^{\circ }$ C for 4 h. Agarose gel electrophoresis was used to analyze the digested products. The agarose gel concentration was 1 %.

### 2.10 Embryo classification

Embryos were graded in accordance with the International Society for Embryo Technology. In brief, the cells were classified as M1 (morula, grade 1: embryos with single or small extruded blastomeres comprising less than 15 % of the total cellular material), M2 (morula, grade 2: large cells or individual blastomeres extruded from the embryonic mass that make up more than 15 % but less than 50 % of the total cellular material), or blastocoel. The embryos with blastomeres containing nuclei, but too underdeveloped to be considered viable, were classified as degenerate. The ovum was designated as being unfertilized when no indication of cleavage was found or when all cytoplasmic fragments lacked a nucleus.

### 2.11 Statistical analysis

The associations between ZNF33B genotypes and superovulation traits were analyzed using the general linear model procedure of SPSS version 16.0. The linear model is as follows:

$$Y_{ijk}=\mu +P_{i}+M_{j}+G_{k}+E_{ijk},$$

where $Y_{ijk}$ is the observation for superovulation traits, μ is the overall population mean, $P_{i}$ is the fixed effect due to the i th parity, $M_{j}$ is the fixed effect of j months of age, $G_{k}$ is the fixed effect associated with k th genotype (AA, AB, and BB genotypes), and $E_{ijk}$ is random error. The significance of differences was tested using Duncan's multiple comparisons.

The experimental data from more than three independent experiments per group were analyzed using the ANOVA module of SPSS version 16.0. The data were expressed as the mean ± standard deviation. Statistical significance was set at p<0.05 .

**Figure 2:**
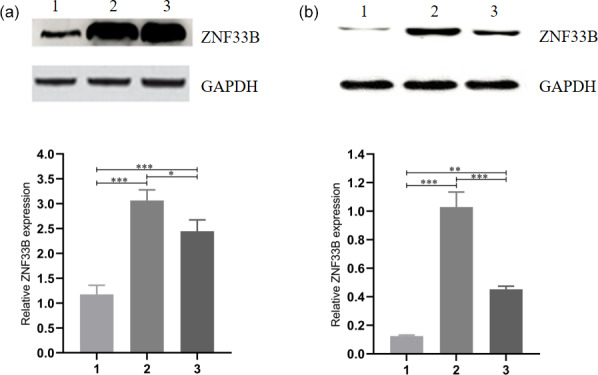
Analysis of the expression of ZNF33B in different periods of bovine ovary and corpus luteum. **(a)** Relative expression of ZNF33B in bovine ovary. (1) Ovarian cortex during corpus phase; (2) ovarian cortex in the follicular phase; (3) ovarian medulla. **(b)** Relative expression of ZNF33B in different stages of corpus luteum. (1) Corpus hemorrhagicum; (2) corpus luteum; (3) corpus albicans. ${}^{*}$ p<0.05 , ${}^{**}$ p<0.01 , ${}^{***}$ p<0.001 .

**Table 2:** Statistical table of cattle superovulation with the different genotypes.

Superovulation traits	Genotypes AA	Genotypes AB	Genotypes BB
NTE	9.64 ± 0.24 ${}^{A}$	13.73 ± 0.53 ${}^{B}$	15.96 ± 0.30 ${}^{C}$
NAE	5.62 ± 0.23 ${}^{A}$	8.18 ± 0.31 ${}^{B}$	8.97 ± 0.09 ${}^{B}$
NDE	2.28 ± 0.04 ${}^{a}$	2.67 ± 0.11 ${}^{a}$	3.41 ± 0.09 ${}^{b}$
NUE	1.74 ± 0.11 ${}^{A}$	2.88 ± 0.17 ${}^{b}$	3.58 ± 0.19 ${}^{C}$

Superovulation traits correspond to the stereomicroscope observation. NUE indicates the number of unfertilized embryos; NDE indicates the number of degenerated embryos; NAE indicates the number of available embryos; NTE indicates the number of total embryos. Values with different lowercase letter superscripts in the same column differ significantly ( p<0.05 ). Values with different uppercase letter superscripts in the same column differ significantly ( p<0.01 ).

## 3 Results

### 3.1 Identification of recently duplicated genes in KRAB-ZNFs during oocyte development

In primates, 19 recently duplicated KRAB-ZNF genes were found. The expression of these genes in human oocyte expression profiles (GSE12034) was characterized, and 10 recently duplicated KRAB-ZNF genes were isolated. The expression of ZNF33B was found to be significantly decreased in human oocytes (Fig. 1a). The transcriptomic data of bovine embryonic developmental stages (GSE143848) showed that ZNF33B was consistently poorly expressed at the oocyte and 2–16 cell stages, but the expression was gradually upregulated. After entering the mulberry embryo and blastocyst stage, ZNF33B was in a high expression state (Fig. 1b).

**Figure 3:**
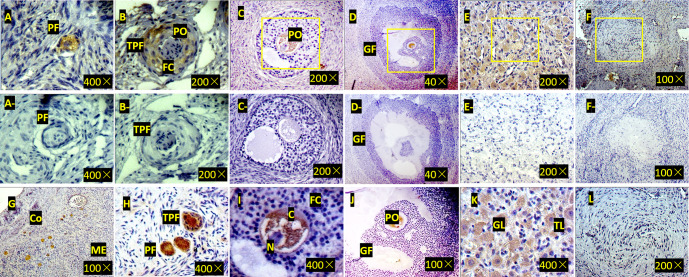
Expression of ZNF33B in bovine ovary **(a–l)**. **(a)** Expression of ZNF33B in primordial follicle tissue. PF is the primordial follicle. **(b)** Expression of ZNF33B in primary follicle tissue. TPF indicates primary follicles, PO indicates primary oocytes, and FC indicates follicular cells. **(c)** Expression of ZNF33B in secondary follicle tissue. **(d)** Expression of ZNF33B in mature follicle tissue. GF indicates mature follicles. **(e)** Expression of ZNF33B in corpus luteum. **(f)** Expression of ZNF33B in corpus albicans. A negative control chart is shown for panels **(a–f)**. **(g)** Expression of ZNF33B in the ovarian cortex. Co is the ovarian cortex, and ME is the ovarian medulla. **(h–l)** Enlarged picture of the indicated part of **(g)** and **(c–f)**. N is nucleus, C is cytoplasm, FC is follicular cells, GL is granulosa luteal cells, and TL is membrane luteal cells. Bar: 20 µ m.

**Figure 4:**
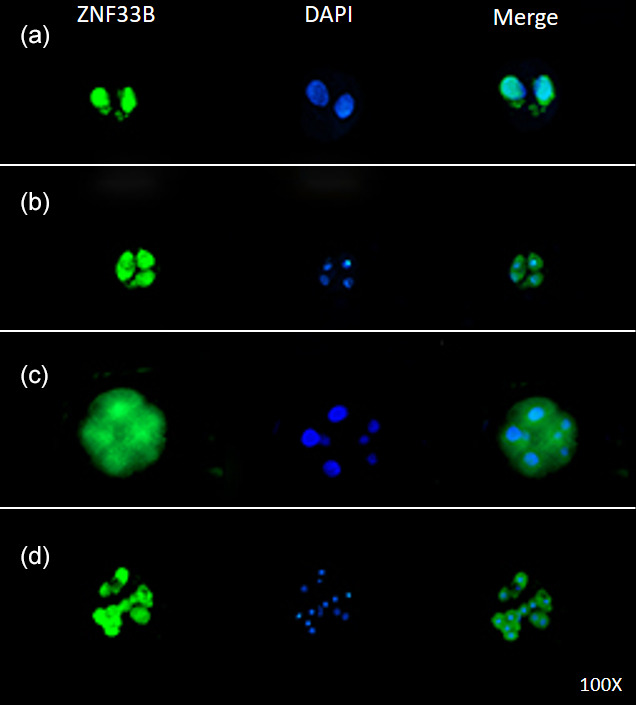
Expression of ZNF33B in bovine early embryos. **(a–d)** Expression of ZNF33B in 2-, 4-, 8-, and 16-cell embryos, respectively.

### 3.2 Relative expression of ZNF33B in cattle ovary

For comparison of the differences in the expression levels between ZNF33B in bovine at the cortical part of the ovary in the luteal-phase ovary, the cortical part of the ovary in the follicular phase ovary, and the medullary part of the ovary, the ZNF33B protein in these three tissues were detected by Western blot. The results showed that ZNF33B was expressed in ovarian cortex of luteal-phase ovary, ovarian cortex of follicular phase ovary, and ovarian medulla. The expression of ZNF33B in ovarian cortex of follicular phase ovaries and medulla was extremely significantly higher than that in ovarian cortex tissue of the luteal-phase ovary (Fig. 2a).

Then, the expression of ZNF33B in corpus hemorrhagicum, corpus luteum, and corpus albicans was detected. The results showed that the expression of ZNF33B was high in the corpus hemorrhagicum and low in the corpus luteum. The expression of ZNF33B was the lowest in the corpus albicans phase. It showed a downward trend during the process from follicular ovulation to luteal degeneration. It was also significantly different at the three stages of the corpus luteum (Fig. 2b).

### 3.3 Localization of ZNF33B in cattle ovary and early embryo

Immunohistochemistry demonstrated that ZNF33B was mainly expressed in the cytoplasm of oocytes and granulosa luteal cells of the ovarian corpus luteum. It was also expressed in the cytoplasm of ovarian stromal cells, cumulus cells, and membranous luteal cells but not in the nucleus of oocytes, cumulus cells, stromal cells, granulosa luteal cells, and membranous luteal cells and corpus albicans (Fig. 3).

Immunofluorescent staining was performed on the early bovine embryos at the 2-, 4-, 8-, and 16-cell stages. The results showed that ZNF33B was expressed at all stages of embryonic development and mainly expressed in the cell nucleus at the two-cell stage. Interestingly, ZNF33B appeared to be expressed throughout the blastomeres at the four-cell stage and early embryonic development after that (Fig. 4).

**Figure 5:**
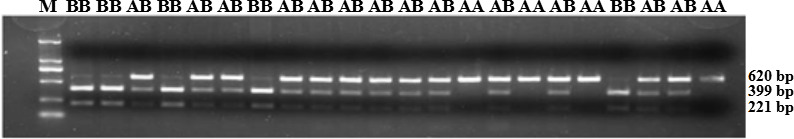
PCR-restriction fragment length polymorphism results for the ZNF33B gene in Changbaishan black cattle. Genotype AA represents allele gene GG, and genotype BB represents allele gene TT, AB for GT or TG. Genotype AA has single band with a length of 620 bp, and BB has double bands (399 and 221 bp); AB has three bands (top to bottom: 620, 399, and 221 bp).

### 3.4 SNP prediction and genotypes

A total of 77 single nucleotide polymorphism (SNP) loci of ZNF33B were found by the National Center for Biotechnology Information (NCBI) SNP database (http://www.ncbi.nlm.nih.gov/snp/?term=, last access: 15 May 2021). After analysis was conducted, the g.-61bpG > T loci in the 5 ${}^{\prime }$ -untranslated region (5 ${}^{\prime }$ UTR) was selected to study the effect of ZNF33B polymorphism with superovulation traits.

ZNF33B gene fragments were amplified from the genomic DNA of all samples. In accordance with the three-band patterns observed after the digestion reaction, the cattle were classified into three groups: AA, AB, and BB. As shown in Fig. 5, the DNA restriction fragments at loci g.-61bpG > T were generated by the ZNF33B polymorphisms: 620 bp for the AA genotype (56 cattle); 399 and 221 bp for the BB genotype (224 cattle); and 221, 399, and 620 bp for the AB genotype (276 cattle). After counting, the frequencies of G and T alleles were found to be 0.3489 and 0.6511, respectively.

### 3.5 Associations of genotypes with superovulation traits

The association analysis between ZNF33B genotypes and superovulation traits is shown in Table 2. The three genotypes showed significant difference in the number of available embryos, unfertilized embryos, and total embryos. The BB genotype had a greater number of total embryos than the AA genotype (15.96 ± 0.30 versus 9.64 ± 0.24; p<0.01 ).

## 4 Discussion

Acquisition of oocyte competence relies on the well-controlled events accompanying follicular development (Albertini et al., 2003; Barret and Albertini, 2010). Multiple ovulations in one reproductive cycle are rare in singleton animals, such as humans, cattle, and sheep. Under the precise regulation of pituitary gonadotropins and several unknown factors, only one follicle is usually able to develop to maturity for ovulation in the natural state, and the only method to obtain sufficient oocytes in a short period of time is to use superovulation (Vieira et al., 2014). During ovarian superovulation, not all oocytes expelled are fully mature. Due to the effects of gonadotropins, phenomena such as aneuploid chromosomes (Munne et al., 1997), immune responses (Shimada et al., 2006), and changes in the expression levels of some genes that regulate development (De Los Santos et al., 2012) may cause oocytes to lose their potential to develop into embryos. Therefore, research has focused on how to obtain quality oocytes for superovulation.

The developmental capacity of oocytes and embryos is associated with subtle changes in the transcriptional profile of certain genes (Nemcova et al., 2016). In addition, the function of transcriptional regulation may be altered by point mutations in the regulatory regions of genes that alter the configuration (Mayo et al., 2006). The A > G mutation was found at exon 1 of bovine inhibin alpha (INHA) at locus 192, and GG individuals were significantly higher than AG and AA individuals in terms of the total number of eggs and higher than AG or AA individuals in terms of the number of embryos that could be transferred; therefore, the mutant AA type was not sensitive to the superovulation response and superovulation was not necessary (Tang et al., 2011). Similarly, two mutations on the progesterone receptor (PRG) have been shown to be associated with the supernumerary ovulation trait, and PRG is thus considered to be a predictive target for the supernumerary ovulation trait in Holstein cows (Yang et al., 2011).

The expression profile analysis of human oocytes showed that the expression of ZNF33B was significantly reduced in the 10 recently duplicated genes. Transcriptome analysis of bovine oocytes showed that the expression of ZNF33B was significantly reduced in oocytes and early embryos before the 16-cell stage, suggesting that ZNF33B may have a potential negative regulation of oocyte maturation and early embryo development. Analysis of the expression of ZNF33B in the ovary showed that the expression of ZNF33B in luteal tissue decreased with the periodic development of the corpus luteum. Further immunohistochemical analysis revealed that ZNF33B was mainly distributed in the cytoplasm of oocytes and granular luteal cells. Immunofluorescence analysis showed that ZNF33B was mainly expressed in the nucleus of the two-cell phase and the whole cell after the two-cell phase. These results suggested that the expression of ZNF33B may have some spatial and temporal specificity. The temporal and spatial specificity of the expression of KRAB-type zinc finger protein is not uncommon. For example, ZNF382 is a zinc finger protein containing nine C ${}_{2}$ H ${}_{2}$ -type zinc fingers at the C terminal of the KRAB (AB) domain, and it is expressed in human early embryos but only in heart tissues at the adult stage (Luo et al., 2002).

In this study, 556 Changbaishan black cattle oocytes were collected after superovulation and analyzed in accordance with the polymorphism of g.-61bpG > T site. The frequencies of G and T alleles were found to be 0.3489 and 0.6511, respectively. The type of oocyte quality statistics found after BB cow individuals in the total number of eggs and number of available embryos was significantly higher than that of AA-type individuals. The results showed that BB-type bovine had a better response to superovulation and could produce more oocytes, while the AA-type bovine had a poor response to superovulation, indicating that ZNF33B may have a potential effect on oocyte maturation, ovulation, and follicular development. The g.-61bpG > T site of ZNF33B gene could be used as a molecular genetic marker for superovulation traits in cattle, and it could guide the breeding and superovulation of cattle.

## 5 Conclusions

In conclusion, ZNf33B g.-61bpG > T mutation is associated with superovulation traits. After superovulation treatment, the cows with T allele of g.-61bpG > T were superior to those with the G allele in terms of ovulation number and available embryos. This study also determined the temporal and spatial specificity of ZNF33B expression in ovaries and early embryos. The results suggested that the polymorphism of ZNF33B could be used as a molecular marker to improve the superovulation performance in cattle.

## Data Availability

The data are available from the corresponding author upon request.
